# Supplementary material for: Identification of photoperiod-induced specific miRNAs in the adrenal glands of Sunite sheep (Ovis aries)
Source: Front Vet Sci. 2022 Jul 22;9:888207. doi: 10.3389/fvets.2022.888207 (PMC9354845; doi:10.3389/fvets.2022.888207)
Supplement: Supplementary file 1 [file Data_Sheet_1.ZIP › Supplementary file/miRDeep2 file.zip!/miRDeep2 file/Novel_Structure/SPLP42A3/Novel_337.pdf]

Provisional ID	:	NC_019458.2_1023
Score total	:	2.3
Score for star read(s)	:	-1.3
Score for read counts	:	0
Score for mfe	:	2
Score for randfold	:	1.6
Score for cons. seed	:	
Total read count	:	1082
Mature read count	:	1082
Loop read count	:	0
Star read count	:	0

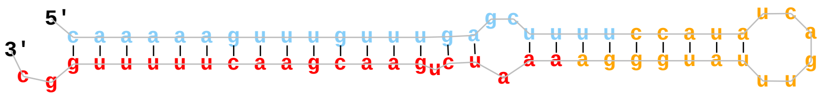

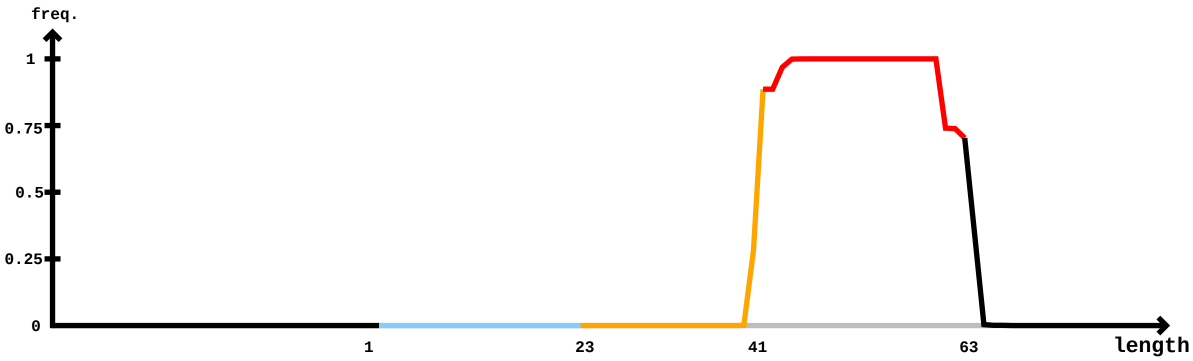


```
                                                   Star                                        Mature
5'- cugauacacagccuugcuaauauuggguuggccaaaaaguuuguuugagcuuuuccauaucaguuuaugggaaaaucugaacgaacuuuuuggccaaugcagugcuauuuuuau   -3'    exp
    .........(((...)))..(((((.((((((((((((((((((((..(((((((((((......))))))))).)).)))))))))))))))))))).))))).........    reads     mm       sample
    ......................................................................gaaaaucugaacgaacuuuuuggc....................    1         0        seq
    .......................................................................aaaaucugaacAaacuuuuu.......................    18        1        seq
    .......................................................................aaaaucugaaUgaacuuuuu.......................    247       1        seq
    .......................................................................aaaaucugaacgaacuuuuu.......................    13        0        seq
    .......................................................................aaaaucugaacgaacuuuuug......................    2         0        seq
    .......................................................................aaaaucugaacAaacuuuuugg.....................    19        1        seq
    .......................................................................aaaaucugaacgaacuuuuugg.....................    5         0        seq
    ........................................................................aaaucugaacAaacuuuuu.......................    3         1        seq
    ........................................................................aaaucugaacAaacuuuuugg.....................    14        1        seq
    ........................................................................aaaucugaacAaacuuuuuggc....................    637       1        seq
    ..........................................................................aucugaacAaacuuuuuggc....................    16        1        seq
    ..........................................................................aucugaacAaacuuuuuggcc...................    73        1        seq
    ...........................................................................ucugaacAaacuuuuuggcc...................    30        1        seq
    ...........................................................................ucugaacAaacuuuuuggcca..................    3         1        seq
    ............................................................................cugaacgaacuuuuuggccaau................    1         0        seq
```
